# Supplementary material for: The role of software in science: a knowledge graph-based analysis of software mentions in PubMed Central
Source: PeerJ Comput Sci. 2022 Jan 14;8:e835. doi: 10.7717/peerj-cs.835 (PMC8771769; doi:10.7717/peerj-cs.835)
Supplement: Supplemental Information 1 [file peerj-cs-08-835-s001.pdf]

Supplementary Material

The Role of Software in Science: A Knowledge Graph-based Analysis of Software Mentions in PubMed Central

David Schindler[1], Felix Bensmann[2], Stefan Dietze[2,3], and Frank Krüger[1,4]

[1]Institute of Communications Engineering, University of Rostock, Germany
[2]GESIS - Leibniz Institute for the Social Sciences, Cologne, Germany
[3]Heinrich-Heine-University Düsseldorf, Germany
[4]Department Knowledge, Culture & Transformation, University of Rostock, Germany

	Wikipedia-PubMed Word2Vec ($M_{L,sw,-}$)					
Trainable	Precision		Recall		FScore	
	Test	Devel	Test	Devel	Test	Devel
False	0.853±0.013	0.815±0.02	0.799±0.023	0.745±0.026	0.825±0.007	0.778±0.007
True	0.881±0.009	0.842±0.018	0.786±0.021	0.722±0.008	0.831±0.011	0.777±0.005

Table A1: Overview of hyper-parameter results for using a Bi-LSTM-CRF ($M_{L,sw,-}$) with pre-trained word embedding established by Pyysalo et al. (2013). The word embedding is either trained with the model (True) or frozen while training the model (False).

	Custom Embedding ($M_{L,sw,-}$)					
Trainable	Precision		Recall		FScore	
	Test	Devel	Test	Devel	Test	Devel
False	0.863±0.018	0.821±0.017	0.814±0.006	0.764±0.014	0.838±0.01	0.791±0.006
True	0.866±0.017	0.831±0.02	0.797±0.01	0.752±0.015	0.83±0.008	0.789±0.004

Table A2: Overview of hyper-parameter results for using a Bi-LSTM-CRF ($M_{L,sw,-}$) with a custom Word2Vec (Mikolov et al., 2013) model trained on all available articles from PMC OA. The word embedding is either trained with the model (True) or frozen while training the model (False).

	LSTM Size ($M_{L,sw,-}$)					
LSTM size	Precision		Recall		FScore	
	Test	Devel	Test	Devel	Test	Devel
100	0.864±0.019	0.831±0.017	0.806±0.01	0.752±0.011	0.834±0.008	0.789±0.003
50	0.852±0.02	0.831±0.02	0.785±0.016	0.751±0.023	0.816±0.003	0.788±0.007

Table A3: Overview of results for tuning the size of the Bi-LSTM-CRF model.

Downsampling negative sentences ($M_{L,sw,-}$)						
Downsampling	Precision		Recall		FScore	
	Test	Devel	Test	Devel	Test	Devel
.75	0.855±0.022	0.821±0.028	0.82±0.018	0.765±0.015	0.836±0.003	0.791±0.006
.50	0.832±0.03	0.817±0.022	0.804±0.025	0.756±0.017	0.817±0.017	0.785±0.002

Table A4: Overview of results for Bi-LSTM-CRF model with respect to downsampling of negative sentences containing no software entities, which leads to a higher relative frequency of software in the training set. Provided numbers indicate the percentage of remaining overall negative samples.

Character dim ($M_{L,sw,-}$)						
Layer size	Precision		Recall		FScore	
	Test	Devel	Test	Devel	Test	Devel
10-5	0.852±0.023	0.816±0.022	0.805±0.008	0.764±0.006	0.828±0.013	0.789±0.013
10-10	0.854±0.013	0.836±0.015	0.791±0.007	0.75±0.004	0.821±0.006	0.79±0.007
10-20	0.87±0.015	0.827±0.009	0.797±0.014	0.758±0.015	0.832±0.006	0.79±0.004
10-30	0.837±0.018	0.812±0.013	0.81±0.017	0.769±0.01	0.823±0.006	0.79±0.003
25-10	0.863±0.019	0.829±0.016	0.813±0.013	0.762±0.011	0.837±0.008	0.794±0.004
50-10	0.879±0.003	0.841±0.007	0.791±0.004	0.747±0.009	0.833±0.003	0.791±0.008

Table A5: Overview of results for Bi-LSTM-CRF model with varying layer size for character feature generation. The first number refers to the size of the character embedding and the second to the size of the character LSTM.

Adding Custom features ($M_{L,sw,-}$)						
Custom features	Precision		Recall		FScore	
	Test	Devel	Test	Devel	Test	Devel
with	0.857±0.024	0.828±0.015	0.816±0.013	0.757±0.021	0.836±0.01	0.79±0.005
without	0.866±0.017	0.831±0.02	0.797±0.01	0.752±0.015	0.83±0.008	0.789±0.004

Table A6: Overview of results for Bi-LSTM-CRF model if custom rules are included as features. Rules take distant supervision and string based features into account, their implementation details can be found the corresponding code. *with*: using custom features, *without*: not using custom features.

Downsampling ($M_{SB,sw,-}$)						
Downsampling	Precision		Recall		FScore	
	Test	Devel	Test	Devel	Test	Devel
None	0.87±0.009	0.863±0.016	0.879±0.006	0.844±0.009	0.874±0.007	0.853±0.003
.75	0.871±0.016	0.85±0.015	0.899±0.011	0.871±0.011	0.885±0.007	0.86±0.006
.50	0.869±0.018	0.854±0.006	0.882±0.005	0.846±0.008	0.876±0.009	0.85±0.002

Table A7: Overview of results for downsampling with SciBERT model with respect to downsampling of negative sentences containing no software entities, which leads to a higher relative frequency of software in the training set. Provided numbers indicate the percentage of remaining overall negative samples.

Dropout	Dropout ($M_{SB,sw,-}$) Precision		Recall		FScore	
	Test	Devel	Test	Devel	Test	Devel
0.0	0.87±0.009	0.863±0.016	0.879±0.006	0.844±0.009	0.874±0.007	0.853±0.003
.20	0.888±0.013	0.865±0.01	0.898±0.012	0.86±0.009	0.893±0.01	0.863±0.005
.30	0.881±0.013	0.86±0.014	0.891±0.011	0.865±0.008	0.886±0.011	0.862±0.004

Table A8: Overview of applying dropouts to SciBERT fine-tuning. Values provide the percentage of performed dropouts.

Learning rate	LR ($M_{SB,sw,-}$) Precision		Recall		FScore	
	Test	Devel	Test	Devel	Test	Devel
1e−5	0.873±0.009	0.856±0.006	0.9±0.006	0.861±0.01	0.886±0.004	0.859±0.005
5e−5	0.884±0.008	0.868±0.006	0.897±0.011	0.865±0.012	0.89±0.004	0.866±0.008
1e−6	0.854±0.013	0.837±0.011	0.902±0.016	0.865±0.013	0.877±0.004	0.851±0.003
5e−6	0.849±0.012	0.827±0.008	0.909±0.014	0.871±0.013	0.878±0.011	0.849±0.01

Table A9: Overview of adjusting the learning rate for SciBERT fine-tuning.

Gradient clipping	Gradient Clipping ($M_{SB,sw,-}$) Precision		Recall		FScore	
	Test	Devel	Test	Devel	Test	Devel
1.0	0.87±0.009	0.863±0.016	0.879±0.006	0.844±0.009	0.874±0.007	0.853±0.003
2.0	0.873±0.004	0.844±0.011	0.885±0.009	0.857±0.013	0.879±0.004	0.85±0.002
3.0	0.866±0.007	0.847±0.008	0.888±0.02	0.849±0.013	0.877±0.007	0.848±0.005

Table A10: Overview of performing gradient clipping at different thresholds for SciBERT fine-tuning.

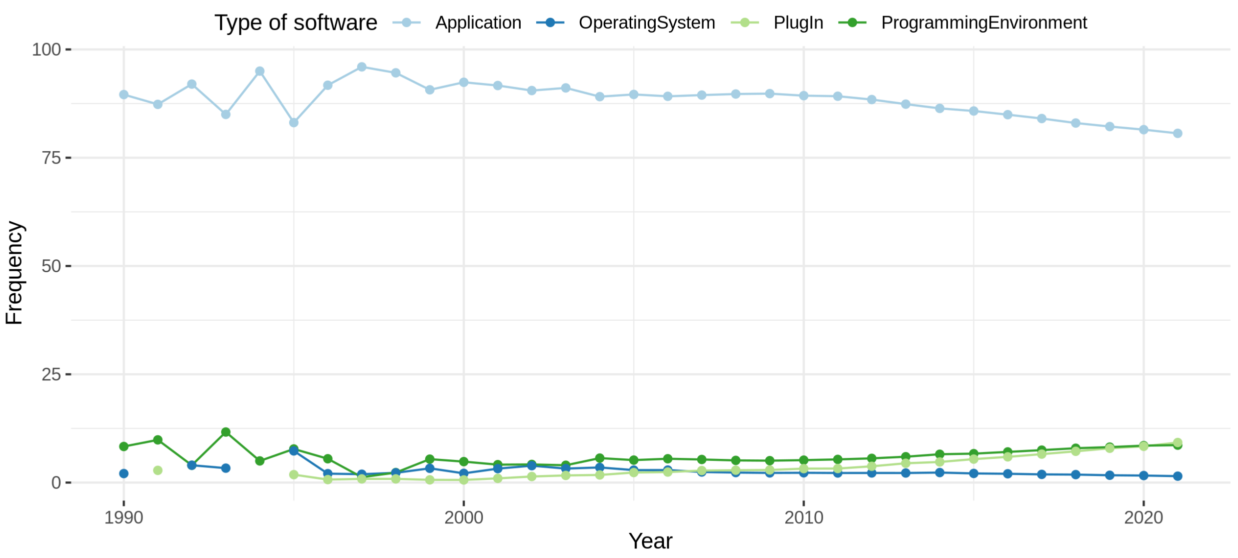

Figure A1: Relative frequencies of software types per year.

Main research domain	# Articles	# Journals
Medicine	1,938,910	4,455
Biochemistry, Genetics and Molecular Biology	1,039,046	1,560
Agricultural and Biological Sciences	416,495	743
Immunology and Microbiology	266,712	451
Chemistry	213,184	407
Neuroscience	175,350	451
Multidisciplinary	161,532	26
Pharmacology, Toxicology and Pharmaceutics	148,307	483
Physics and Astronomy	118,655	332
Environmental Science	101,434	462
Materials Science	92,072	341
Chemical Engineering	88,722	234
Computer Science	77,037	396
Engineering	67,345	501
Psychology	49,655	561
Nursing	47,630	355
Social Sciences	46,730	1,136
Mathematics	39,220	364
Health Professions	36,622	286
Veterinary	29,924	104
Dentistry	25,146	103
Arts and Humanities	8,756	469
Energy	4,105	84
Earth and Planetary Sciences	4,025	247
Business, Management and Accounting	2,574	173
Decision Sciences	2,507	100
Economics, Econometrics and Finance	2,178	181

Table A11: Overview of the number of journals and articles per main research domain. Note that both journals and articles may have multiple categories.

References

Mikolov, T., Chen, K., Corrado, G., and Dean, J. (2013). Efficient estimation of word representations in vector space. *arXiv preprint arXiv:1301.3781*.

Pyysalo, S., Ginter, F., Moen, H., Salakoski, T., and Ananiadou, S. (2013). Distributional semantics resources for biomedical text processing. In *Proceedings of the 5th International Symposium on Languages in Biology and Medicine*, pages 39–44.
